# Do We Overestimate the Within-Variability? The Impact of Measurement Error on Intraclass Coefficient Estimation

**DOI:** 10.3389/fpsyg.2020.00825

**Published:** 2020-05-19

**Authors:** Rafael Wilms, Ralf Lanwehr, Andreas Kastenmüller

**Affiliations:** ^1^Department of Education Studies and Psychology, University of Siegen, Siegen, Germany; ^2^Department of International Management, South Westphalia University of Applied Sciences, Meschede, Germany

**Keywords:** intraclass coefficient, measurement error, within-variance, between-variance, reliability

## Abstract

Many psychological phenomena have a multilevel structure (e.g., individuals within teams or events within individuals). In these cases, the proportion of between-variance to total-variance (i.e., the sum between-variance and within-variance) is of special importance and usually estimated by the intraclass coefficient (1)^1^ [ICC(1)]. Our contribution firstly shows via mathematical proof that measurement error increases the within-variance, which in turn decreases the ICC(1). Further, we provide a numerical example, and examine the RMSEs, alpha error rates and the inclusion of zero in the confidence intervals for ICC(1) estimation with and without measurement error. Secondly, we propose two corrections [i.e., the reliability-adjusted ICC(1) and the measurement model-based ICC(1)] that yield correct estimates for the ICC(1), and prove that they are unaffected by measurement error mathematically. Finally, we discuss our findings, point out examples of the underestimation of the ICC(1) in the literature, and reinterpret the results of these examples in the light of our new estimator. We also illustrate the potential application of our work to other ICCs. Finally, we conclude that measurement error distorts the ICC(1) to a non-negligible extent.

## Introduction

Many psychological phenomena have a multilevel structure (e.g., Nezlek, 2001, 2008; Fleeson, 2004, 2017); observations on a micro-level are nested in a macro-level (e.g., individuals within teams or events within individuals). The intraclass coefficient (1)^1^ (ICC(1)) reflects the degree of resemblance of micro-level units (e.g., events) within macro-level units (e.g., individuals), and is calculated as between-variance (e.g., variance, existing *between* groups or individuals) divided by total-variance (e.g., the sum of between-variance and the variance existing *within* groups or individuals; e.g., Fisher, 1934; Shrout and Fleiss, 1979; Snijders and Bosker, 2012). It is often used to provide insights of the magnitude of variance on different levels to test and inform psychological theories (e.g., Bliese et al., 2002; Castro, 2002; Nook et al., 2018; Kivlighan et al., 2019; Podsakoff et al., 2019). For example, Podsakoff et al. (2019) showed that many psychological constructs (e.g., personality, coping, or job characteristics) vary substantially within-person (at least 40%), even though they “have historically been treated as between-person phenomena” (Podsakoff et al., 2019, p. 737). Hence, Kivlighan et al. (2019) showed that group therapy members *mutually influence* their group posttreatment outcomes (e.g., depression, etc.), and found that group membership explains about 6% of variance [ICC(1) = 0.06; Cohen’s *d* of about 0.47].

However, the estimate of the ICC(1) is more informative, if its bias is small or zero [i.e., the estimated ICC(1) is close to the population parameter of the ICC(1)]. Therefore, researchers examined the behavior of the ICC(1) estimator under different scenarios, such as different numbers of groups and group sizes (Bliese and Halverson, 1998), varying numerical value of the population ICC(1), equal or unequal group sizes (Shieh, 2012), different missing value patterns (Newman and Sin, 2009) or varying number of response options in the Likert-type scale (Beal and Dawson, 2007).

We aim to address an additional factor. In psychology, variables or constructs are almost never measured without measurement error (e.g., Lord and Novick, 2008), and the presence of measurement error increases the variable’s variance (e.g., Ree and Carretta, 2006). We argue that measurement error represents a largely overlooked factor that induces bias in ICC(1) estimation. In particular, we aim to show that measurement error does not affect the between-variance, but increases the within-variance. It therefore induces a downward bias in ICC(1) estimation. We provide a numerical example that quantifies the severity of bias under different numerical values of the population ICC(1); level 1 and level 2 sample sizes; and different reliabilities^2^, and examine the RMSEs, alpha error rates and the inclusion of zero in the confidence intervals for ICC(1) estimation with and without measurement error. As our main contribution, we propose two correction-procedures that yield estimates robust to measurement error: The reliability-adjusted ICC(1) and the measurement model-based ICC(1). These ICC(1) estimators allow a correct interpretation of the proportion of between-variance to total-variance, as they remain unbiased with increasing measurement error.

The article is structured in five main sections. The first section introduces the ICC(1). The second section proves that measurement error increases the estimated within-variance, which in turn decreases the ICC(1) expression, and provides a numerical example. The third section proves that an unbiased ICC(1) can be recovered, when the measurement error-affected within-variance is multiplied by the construct’s reliability, whereas the fourth section concerns the estimation of an unbiased ICC(1), relying on a measurement model. Finally, we discuss the study’s implications, limitations and provide directions for future research.

## Intraclass Coefficient(1)

Data often have a hierarchical or clustered structure (e.g., occasions nested in individuals, individuals nested in groups, groups nested in organizations or in general terms micro-units nested in macro-units). The ICC(1) represents the ratio of between-variance to total-variance (i.e., the sum of the macro-unit variance and the micro-unit variance)^3^ and assumes values between zero and one. The closer the value to one, the more variance on the macro-level. The closer the value to zero, the more variance on the micro-level (e.g., Shrout and Fleiss, 1979; Commenges and Jacqmin, 1994; McGraw and Wong, 1996). Throughout the article, macro-units and micro-units refer to individuals and occasions, respectively, for the purpose of illustration. Consequently, we use the terms between-variance (i.e., macro-unit variance) and within-variance (i.e., micro-unit variance) from now on.

An exemplary random variable *y*_*ij*_ of interest (see Eq. 1) can be defined by a constant, *γ*_00_, the individual effect, *U*_*j*_ (that creates between-variance), and the occasion effect, *u*_*ij*_ (that creates within-variance).

(1) $$y_{i\,j}=\gamma_{00}+U_{j}+u_{i\,j}\;i=1,\ldots ,m\;j=1,\ldots ,n$$

The random variables, *U*_*j*_ and *u*_*ij*_, are independent, and normally distributed with a mean of zero and variances of $\sigma_{b\,\left(e\,t\,w\,e\,e\,n\right)}^{2}$ and $\sigma_{w\,\left(i\,t\,h\,i\,n\right)}^{2}$, respectively. Furthermore, we assume equal sample sizes for the observations within individuals (for formulas for unequal group sizes, see for example Snijders and Bosker, 2012). The population ICC(1) is defined as:

(2) $$\rho \,\left(y\right)=\frac{\sigma_{b}^{2}}{\sigma_{b}^{2}+\sigma_{w}^{2}}$$

where $\sigma_{b}^{2}$ and $\sigma_{w}^{2}$ denote the population between-variance and population within-variance, respectively (Fisher, 1934; Shrout and Fleiss, 1979; McGraw and Wong, 1996). In the sample, the estimated within-variance, ${\hat{\sigma }}_{w}^{2}$, is defined by:

(3) $${\hat{\sigma }}_{w}^{2}=\frac{1}{n\,m-n}\,\sum_{j=1}^{n}\sum_{i=1}^{m}{\left(y_{i\,j}-{\bar{y}}_{j}\right)}^{2}$$

where *n* represents the number of different individuals observed (the sum of unique *j*s), *m* represents the number of observed occasions per individual, and $\bar{y}$_*j*_ represents the person mean (for the definitions of the means, see the Supplementary Appendix 1).

The between-variance, $\sigma_{b}^{2}$, can be estimated in the sample by:

(4) $$S_{b}^{2}=\frac{1}{n-1}\,\sum_{j=1}^{n}{\left({\bar{y}}_{j}-\overset{=}{y}\right)}^{2}$$

where $\overset{=}{y}$ represents the grand mean (for the definitions of the means, see the Supplementary Appendix 1).

However, $S_{b}^{2}$ is biased because of sampling error (according to the law of summing normally distributed variances; see for a review Searle, 1995). A correct estimate for $\sigma_{b}^{2}$ is

(5) $${\hat{\sigma }}_{b}^{2}=S_{b}^{2}-\frac{{\hat{\sigma }}_{w}^{2}}{m}$$

In general, variances corrected for sampling error can become negative. For example, if the $S_{b}^{2}$ is small, the ${\hat{\sigma }}_{w}^{2}$ is large and *m* is small, the corrected between-variance, ${\hat{\sigma }}_{b}^{2}$, likely becomes smaller than zero. This violates the definition of the ICC(1), as it cannot assume negative values. Therefore, many researchers set negative values of the between-variance to zero (Shieh, 2012)^4^.

An estimate for the ICC(1) of the variable *y* is completely defined by:

(6) $$\hat{\rho }\,\left(y\right)=\frac{{\hat{\sigma }}_{b}^{2}}{{\hat{\sigma }}_{b}^{2}+{\hat{\sigma }}_{w}^{2}}$$

## The Effect of Measurement Error on the ICC(1)

In this section, we demonstrate that measurement error induces a positive bias in the within-variance estimation that in turn decreases the ICC(1) expression, whereas it does not induce bias in the between-variance. Firstly, we define the random variable *y*_*ij*_ with measurement error as:

(7) $$y_{i\,j}^{{}^{*}}=y_{i\,j}+\varepsilon_{i\,j}$$

where we assume the measurement error, **ε**_*ij*_, to be independently, normally distributed with a mean of zero and a variance of $\sigma_{\varepsilon }^{2}$, satisfying the classical measurement error assumptions (Lord and Novick, 2008). Throughout this article, the star signifies that the variable contains measurement error or that the estimator is applied to a variable with measurement error from now on.

Secondly, and most importantly, we prove that the presence of measurement error leads to an overestimation of the within-variance.

**Theorem 1** Let *y*^∗^ be a random variable, consisting of a random variable *y* with two-levels (e.g., Eq. 1) and some measurement error, ε, then the expectation of the estimator for the within-variance of variable *y*^∗^, σ^w2* (see Eq. 8), equals the sum of the within-variance, $\sigma_{w}^{2}$, and the variance of the measurement error, $\sigma_{\varepsilon }^{2}$.

E⁢(σ^w2*)=σw2+σε2

Theorem 1 highlights that the within-variance estimator applied to a variable, contaminated by measurement error yields biased estimates.

*Proof.* We define σ^w2*, as the estimator of the within-variance for the random variable *y*^∗^:

(8) σ^w2*=1n⁢m-n⁢∑j=1n∑i=1m(yi⁢j*-y¯j)2

which – if we substitute $y_{i\,j}^{{}^{*}}$ with *y*_*ij*_ + **ε**_*ij*_ and expand the product – yields:

(9) σ^w2*=1n⁢m-n⁢∑j=1n∑i=1m((yi⁢j+εi⁢j-y¯j)⁢(yi⁢j+εi⁢j-y¯j))

which can be rewritten as:

(10) σ^w2*=1n⁢m-n⁢∑j=1n∑i=1m(yi⁢j2+εi⁢j2+y¯j2+2⁢yi⁢j⁢εi⁢j-2⁢yi⁢j⁢y¯j-2⁢εi⁢j⁢y¯j)

and

σ^w2*=1n⁢m-n⁢∑j=1n∑i=1m(yi⁢j-y¯j)2+1n⁢m-n⁢∑j=1n∑i=1mεi⁢j2

(11) $$+\frac{1}{n\,m-n}\,\sum_{j=1}^{n}\sum_{i=1}^{m}\left(2\,y_{i\,j}\,\varepsilon_{i\,j}-2\,\varepsilon_{i\,j}\,{\bar{y}}_{j}\right)$$

Eq. 11 proves that applying the standard estimator of the within-variance to a variable, contaminated by measurement error overestimates the within-variance. The standard estimator contains the within-variance of the original variable (without measurement error), expressed by the first summation on the right hand side, but also measurement error-induced bias, expressed by the second and third summation on the right hand side. Taking the expectation of σ^w2* (see Eq. 12), $E\,\left({\hat{\sigma }}_{w}^{2}\right)$ converges to the true within-variance, $E\,\left({\hat{\sigma }}_{\varepsilon }^{2}\right)$ converges to the true variance of the measurement error, and *E*(*residual*) converges to zero, since **ε**_*ij*_ is assumed to be independent of *y*_*ij*_ and $\bar{y}$_*j*_. We conclude that σ^w2* is biased, unless the variance of the measurement error is equal to zero.

(12) E⁢(σ^w2*)=E⁢(σ^w2)+E⁢(σ^ε2)+E⁢(r⁢e⁢s⁢i⁢d⁢u⁢a⁢l)=σw2+σε2

This completes the proof.

Thirdly, we prove that measurement error does not affect the sampling error-corrected between-variance estimator (see Eq. 5). The measurement error does not affect the estimation of averages, such as the grand mean and the person mean (Ree and Carretta, 2006). However, creating the person mean induces sampling error in the between-variance, as the occasions are averaged. The variance of $\bar{y}$${}_{j}^{{}^{*}}$ consists of four parts (see Eq. 13): the constant **γ**_*00*_, the variable *U*_*j*_, the average of the variable *u*_*ij*_, and the average of the variable **ε**_*ij*_.

$${\bar{y}}_{j}^{{}^{*}}=\frac{1}{m}\,\sum_{i=1}^{m}\left(\gamma_{00}+U_{j}+u_{i\,j}+\varepsilon_{i\,j}\right)$$

(13) $$=\gamma_{00}+U_{j}+\frac{1}{m}\,\sum_{i=1}^{m}\left(u_{i\,j}\right)+\frac{1}{m}\,\sum_{i=1}^{m}\left(\varepsilon_{i\,j}\right)$$

As the variables **γ**_*00*_, *U*_*j*_, *u*_*ij*_, and **ε**_*ij*_ are independent, we can write the variance of $\bar{y}$${}_{j}^{{}^{*}}$ as the sum of its components (see Eq. 14).

$$V\,a\,r\,\left({\bar{y}}_{j}^{{}^{*}}\right)=V\,a\,r\,\left(U_{j}\right)+V\,a\,r\,\left(\frac{\sum_{i=1}^{m}u_{i\,j}}{m}\right)+V\,a\,r\,\left(\frac{\sum_{i=1}^{m}\varepsilon_{i\,j}}{m}\right)$$

(14) $$=V\,a\,r\,\left(U_{j}\right)+\frac{V\,a\,r\,\left(u_{i\,j}\right)+V\,a\,r\,\left(\varepsilon_{i\,j}\right)}{m}={\hat{\sigma }}_{b}^{2}+\frac{{\hat{\sigma }}_{w}^{2}+{\hat{\sigma }}_{\varepsilon }^{2}}{m}$$

*Var*(*γ*_00_) is zero. Eq. 14 shows that the variance of $\bar{y}$${}_{j}^{{}^{*}}$ equals the between-variance, ${\hat{\sigma }}_{b}^{2}$, plus the sampling error $\frac{{\hat{\sigma }}_{w}^{2}+{\hat{\sigma }}_{\varepsilon }^{2}}{m}$, which equals σb2+σw2*m (see Theorem 1). As the estimated between-variance (see Eq. 5) is corrected for sampling error, it is unaffected by measurement error.

This completes the proof.

We have shown that measurement error induces bias in the within-variance estimator, but not in the between-variance estimator. Now, we can show the effect of measurement error on ICC(1) estimation. Based on Theorem 1 (based on sample analogs), we derive Corollary 1.

**Corollary 1** Let *y* be a random variable with two levels (e.g., Eq. 1) and let *y*^∗^ be the same variable plus some additional measurement error, ε, then the estimate of the ICC(1) of variable *y*^∗^ is smaller than (or equal to) the estimate of the ICC(1) of variable *y* (if ${\hat{\sigma }}_{\varepsilon }^{2}$ = 0; see Theorem 1).

$$\hat{\rho }\,\left(y^{{}^{*}}\right)=\frac{{\hat{\sigma }}_{b}^{2}}{{\hat{\sigma }}_{b}^{2}+{\hat{\sigma }}_{w}^{2}+{\hat{\sigma }}_{\varepsilon }^{2}}\leq \hat{\rho }\,\left(y\right)=\frac{{\hat{\sigma }}_{b}^{2}}{{\hat{\sigma }}_{b}^{2}+{\hat{\sigma }}_{w}^{2}}$$

Corollary 1 highlights that the ICC(1) estimator underestimates the ICC(1), if applied to a variable with measurement error, as shown by the comparison of $\hat{\rho }\,\left(y^{{}^{*}}\right)$ and $\hat{\rho }\,\left(y\right)$. All in all, our results highlight the need to derive an estimator of the within-variance, robust to measurement error, which in turn allows ICC(1) estimation, robust to measurement error.

### Numerical Example of the Effect of Measurement Error on ICC(1) Estimation

In order to illustrate the effect of measurement error on ICC(1) estimation, we created a numerical example, which compares the ICC(1) estimation with and without measurement error.

Firstly, we examine the performance of ICC(1) estimation, using the root mean square error (RMSE). Secondly, we examine the alpha error rate, using confidence intervals (based on the exact confidence limit equation; Searle, 1997). The alpha error rate is crucial for inference based on ICC(1) estimation. Thirdly, we examine the inclusion of zero in the confidence intervals, which is important as many researchers use the ICC(1) to understand whether or not their data are independent.

#### Setup of the Numerical Examples

We created a numerical example with different setups for sample sizes, different numerical values of the population ICC(1), and reliabilities, which were performed with R Cran (R Core Team, 2015). We defined the data-generating process for the variable $y_{i\,j}^{{}^{*}}$ according to Eq. 7, where *U*_*j*_ and *u*_*ij*_ are drawn from different normal distributions; each with a mean of zero and a different value for the standard-deviation [i.e., equivalent to a population ICC(1) of 0.1, 0.3, 0.5, 0.7, and 0.9]. The constant, **γ**_*00*_, is set to zero. The measurement error is normally distributed with a mean of zero and a standard deviation, equivalent to reliabilities of approximately 1, 0.9, 0.7, and 0.5 for $y_{i\,j}^{{}^{*}}$. Those values reflect no measurement error, substantial reliability, moderate reliability and fair reliability (Shrout, 1998), respectively. Reliability is defined in Eq. 15 later in the manuscript. We use the model with a reliability of 1 as a reference model. We simulated samples from these models for different numbers of individuals and events (i.e., 25, 40; 50, 20; 100, 10; 125, 8; and 250, 4), as ICC(1) estimation has been shown to be sensitive to group sizes (Shieh, 2012). We repeated this process 5.000 times for each of the configurations [i.e., 5 different ICC(1), 3 different reliabilities, and 5 different sample size configurations].

#### Results

Table 1 shows the comparison of the RMSE of ICC(1) estimation without and with measurement error. The accuracy of ICC(1) estimation deteriorates with increasing measurement error. For a reliability of 1, the RMSEs, averaged over the sample configurations range from 0.018 to 0.048. For a reliability of 0.9, the RMSEs, averaged over the sample configurations range from 0.023 to 0.057. For a reliability of 0.7, the RMSEs, averaged over the sample configurations range from 0.037 to 0.104. For a reliability of 0.5, the RMSEs, averaged over the sample configurations range from 0.052 to 0.176. Moreover, the RMSEs of the ICC(1) become larger with decreasing sample sizes on level 2 and increasing sample size on level 1 in the sample configurations for all degrees of measurement error. Moreover, RMSEs become smaller, the more the population ICC(1) diverges from 0.5.

**TABLE 1:** RMSE of ICC(1) estimation without measurement error, $\hat{\rho }$, and with measurement error, $\hat{\rho }$*.

		**25, 40**		**50, 20**		**100, 10**		**125, 8**		**250, 4**	
**ICC(1)**	**Rel**	**$\hat{\rho }$**	$\hat{\rho }$*	**$\hat{\rho }$**	$\hat{\rho }$*	**$\hat{\rho }$**	$\hat{\rho }$*	**$\hat{\rho }$**	$\hat{\rho }$*	**$\hat{\rho }$**	$\hat{\rho }$*
0.9	0.9	0.031	0.037	0.020	0.025	0.014	0.019	0.013	0.018	0.010	0.015
0.9	0.7	0.031	0.060	0.020	0.049	0.014	0.043	0.013	0.042	0.010	0.040
0.9	0.5	0.031	0.104	0.020	0.093	0.014	0.088	0.013	0.087	0.010	0.084
0.7	0.9	0.067	0.077	0.046	0.055	0.034	0.043	0.030	0.040	0.024	0.035
0.7	0.7	0.067	0.117	0.045	0.099	0.034	0.091	0.030	0.089	0.024	0.086
0.7	0.5	0.066	0.187	0.046	0.175	0.033	0.169	0.030	0.168	0.024	0.166
0.5	0.9	0.074	0.082	0.054	0.062	0.042	0.051	0.038	0.048	0.032	0.043
0.5	0.7	0.076	0.121	0.055	0.107	0.041	0.099	0.038	0.098	0.032	0.095
0.5	0.5	0.075	0.186	0.054	0.177	0.041	0.173	0.039	0.172	0.032	0.171
0.3	0.9	0.064	0.067	0.048	0.052	0.038	0.044	0.036	0.042	0.035	0.042
0.3	0.7	0.064	0.092	0.049	0.084	0.039	0.078	0.037	0.078	0.034	0.078
0.3	0.5	0.065	0.134	0.048	0.130	0.038	0.128	0.037	0.128	0.034	0.128
0.1	0.9	0.032	0.032	0.026	0.027	0.026	0.027	0.026	0.027	0.030	0.032
0.1	0.7	0.032	0.039	0.027	0.036	0.025	0.036	0.025	0.036	0.030	0.040
0.1	0.5	0.032	0.052	0.027	0.051	0.026	0.052	0.026	0.052	0.030	0.055

**The term Rel abbreviates the term reliability*.*

Table 2 shows the comparison of the alpha error rate of ICC(1) without and with measurement error. The averaged alpha error rate for ICC(1) without measurement error equals roughly the expected 5%. For the ICC(1) estimation with measurement error, we observe increasing alpha error rates with decreasing reliability. For a reliability of 0.9, the alpha error rates, averaged over the sample configurations equal 6.1 to 9.8%, and is thus higher than 5%. For reliabilities of 0.7, and 0.5, the alpha error rates, averaged over the sample configurations increase further, and range from 16.6 to 56.1%, and 43.3 to 89.4%, respectively. Moreover, the alpha error rate becomes larger with increasing sample size on level 2 and decreasing sample size on level 1 in the sample configurations. Additionally, the higher the numerical value of the population ICC(1), the higher the alpha error rate.

**TABLE 2:** Alpha error rate of ICC(1) estimation without measurement error, $\hat{\rho }$, and with measurement error, $\hat{\rho }$*.

		**25, 40**		**50, 20**		**100, 10**		**125, 8**		**250, 4**	
**ICC(1)**	**Rel**	**$\hat{\rho }$**	$\hat{\rho }$*	**$\hat{\rho }$**	$\hat{\rho }$*	**$\hat{\rho }$**	$\hat{\rho }$*	**$\hat{\rho }$**	$\hat{\rho }$*	**$\hat{\rho }$**	$\hat{\rho }$*
0.9	0.9	0.052	0.061	0.048	0.068	0.050	0.096	0.054	0.112	0.055	0.155
0.9	0.7	0.048	0.189	0.051	0.350	0.053	0.626	0.049	0.722	0.046	0.916
0.9	0.5	0.053	0.571	0.048	0.903	0.048	0.996	0.049	0.999	0.049	1.000
0.7	0.9	0.052	0.058	0.051	0.066	0.051	0.096	0.047	0.099	0.051	0.143
0.7	0.7	0.056	0.183	0.047	0.338	0.053	0.582	0.052	0.670	0.048	0.860
0.7	0.5	0.050	0.558	0.053	0.880	0.051	0.993	0.050	0.998	0.049	1.000
0.5	0.9	0.046	0.052	0.047	0.066	0.052	0.091	0.049	0.101	0.046	0.128
0.5	0.7	0.049	0.173	0.052	0.315	0.047	0.523	0.047	0.589	0.050	0.746
0.5	0.5	0.046	0.546	0.049	0.852	0.048	0.982	0.054	0.990	0.046	0.998
0.3	0.9	0.050	0.061	0.047	0.060	0.046	0.077	0.047	0.081	0.053	0.098
0.3	0.7	0.048	0.170	0.050	0.290	0.052	0.414	0.048	0.474	0.053	0.513
0.3	0.5	0.055	0.495	0.048	0.784	0.048	0.927	0.050	0.948	0.046	0.961
0.1	0.9	0.053	0.053	0.049	0.060	0.056	0.067	0.051	0.064	0.050	0.062
0.1	0.7	0.049	0.137	0.051	0.175	0.046	0.190	0.045	0.178	0.052	0.148
0.1	0.5	0.055	0.352	0.052	0.471	0.054	0.506	0.052	0.479	0.045	0.356

**The term Rel abbreviates the term reliability*.*

Table 3 shows the comparison of the inclusion of zero in the confidence interval of the ICC(1) estimation without and with measurement error. For the inclusion of zero in the confidence interval, only population ICC(1) of 0.1 appears to be of interest. Our analysis reveals that zero is included in confidence interval more often with increasing measurement error. For ICC(1) without measurement error, the confidence intervals included zero – on average over the sample configurations – 1.2% of the times. For a reliability of 0.9, 0.7, and 0.5, the confidence interval of the ICC(1) included zero – on average over the sample configurations – 2.3, 6.7, and 18.8% of the times, respectively. Moreover, the confidence interval includes zero more often with increasing sample size on level 2 and decreasing sample size on level 1 in the sample configurations.

**TABLE 3:** The inclusion of zero in the confidence interval of ICC(1) estimation without measurement error, $\hat{\rho }$, and with measurement error, $\hat{\rho }$*.

		**25, 40**		**50, 20**		**100, 10**		**125, 8**		**250, 4**	
**ICC(1)**	**Rel**	**$\hat{\rho }$**	$\hat{\rho }$*	**$\hat{\rho }$**	$\hat{\rho }$*	**$\hat{\rho }$**	$\hat{\rho }$*	**$\hat{\rho }$**	$\hat{\rho }$*	**$\hat{\rho }$**	$\hat{\rho }$*
0.9	0.9	0.000	0.000	0.000	0.000	0.000	0.000	0.000	0.000	0.000	0.000
0.9	0.7	0.000	0.000	0.000	0.000	0.000	0.000	0.000	0.000	0.000	0.000
0.9	0.5	0.000	0.000	0.000	0.000	0.000	0.000	0.000	0.000	0.000	0.000
0.7	0.9	0.000	0.000	0.000	0.000	0.000	0.000	0.000	0.000	0.000	0.000
0.7	0.7	0.000	0.000	0.000	0.000	0.000	0.000	0.000	0.000	0.000	0.000
0.7	0.5	0.000	0.000	0.000	0.000	0.000	0.000	0.000	0.000	0.000	0.000
0.5	0.9	0.000	0.000	0.000	0.000	0.000	0.000	0.000	0.000	0.000	0.000
0.5	0.7	0.000	0.000	0.000	0.000	0.000	0.000	0.000	0.000	0.000	0.000
0.5	0.5	0.000	0.000	0.000	0.000	0.000	0.000	0.000	0.000	0.000	0.000
0.3	0.9	0.000	0.000	0.000	0.000	0.000	0.000	0.000	0.000	0.000	0.000
0.3	0.7	0.000	0.000	0.000	0.000	0.000	0.000	0.000	0.000	0.000	0.000
0.3	0.5	0.000	0.000	0.000	0.000	0.000	0.000	0.000	0.000	0.000	0.000
0.1	0.9	0.001	0.002	0.000	0.001	0.002	0.005	0.004	0.009	0.054	0.096
0.1	0.7	0.001	0.004	0.000	0.007	0.001	0.030	0.004	0.050	0.055	0.243
0.1	0.5	0.001	0.029	0.000	0.049	0.001	0.145	0.003	0.214	0.055	0.501

**The term Rel abbreviates the term reliability*.*

### Preliminary Discussion of the Effect of Measurement Error on the ICC(1)

We have proven mathematically that measurement error induces a negative bias in ICC(1) estimation (see Corollary 1). In particular, the between-variance remains unbiased even with increasing measurement error. Averaging over the occasion creates sampling error, but the between-variance is corrected for it. Correcting for sampling error automatically corrects for eventual measurement error, if it is corrected by the biased within-variance.

By contrast, measurements error induces positive bias in the within-variance estimation, since it consists of the variable’s actual within-variance and the variance of the measurement error (see Theorem 1). This explains why measurement error induces negative bias in ICC(1) estimation, as the within-variance is part of the denominator. The increase in the denominator reduces the ICC(1) expression.

The numerical example provides an illustration of the effect of measurement error on ICC(1) estimation under various values of the population ICC(1), level 1 and level 2 sample size configurations and reliabilities. The analysis of the RMSE revealed small to severe distortions of the estimated ICC(1), which has already been derived by Corollary 1. The distortion of the ICC(1) becomes larger the more the population ICC(1) diverges from 0.5. Hence, the distortion of the ICC(1) becomes smaller (larger) for larger (smaller) level 2 sample sizes and smaller (larger) level 1 sample sizes in the sample size configurations.

The analysis of the alpha error rate shows that inference based on the ICC(1), affected by the measurement error, is often seriously misleading. In particular, the larger the variance of measurement error, the larger the alpha error rate. The distortion of the alpha error rate becomes smaller (larger) for smaller (larger) population ICC(1). However, the distortion of the alpha error rate becomes smaller (larger) for smaller (larger) level 2 sample sizes and larger (smaller) level 1 sample sizes in the sample configurations. Accordingly, studies with small level 1 sample sizes are more prone to conclude that their data is independent.

The analysis of the inclusion of zero in the confidence interval shows that the ICC(1), affected by measurement error is often not significantly different from zero, even though, the population ICC(1) equals 0.1. The larger the measurement error, the higher the frequency of the inclusion of zero in the confidence interval. In those cases, many researchers may falsely conclude that their data are independent. With increasing level 2 sample size and decreasing level 1 sample size in the sample configurations, the effect becomes more severe.

All in all, our results highlight the need to derive an estimator of the within-variance, robust to measurement error, which in turn allows ICC(1) estimation, robust to measurement error.

## The Reliability-Adjusted ICC(1) Estimator

In this section, we propose a correction for the ICC(1) estimator, based on a construct’s reliability^5^. Firstly, reliability reflects “the degree of true-score variation relative to observed-score variation” (Lord and Novick, 2008, p. 61). In other words, it reflects the degree to which a measure is free from error. In the current context, we can define the population within-reliability (e.g., Raudenbush et al., 1991; Lord and Novick, 2008; Bonito et al., 2012; Nezlek, 2017) as:

(15) $$\alpha =\frac{\sigma_{w}^{2}}{\sigma_{w}^{2}+\sigma_{\varepsilon }^{2}}$$

where $\sigma_{w}^{2}$ refers to the true within-variance of the variable *y*_*ij*_, and $\sigma_{\varepsilon }^{2}$ refers to the variance of the measurement error, **ε**_*ij*_ (Lord and Novick, 2008).

Secondly, it can be shown that the true within-variance, $\sigma_{w}^{2}$, is equivalent to the product of the reliability, α, and the measurement error-affected within-variance, σw2*.

**Theorem 2** Let *y*^∗^ be a random variable, consisting of a random variable *y* with two levels (e.g., Eq. 1) and some measurement error on level 1, ε, then the true value of the within-variance equals the true value of the measurement-affected within-variance multiplied by the true value of the reliability of variable *y*^∗^.

α⁢σw2*=σw2

Theorem 2 highlights that the correct within-variance can be recovered, by using the product of the true value of the measurement error-affected within-variance and the true value of the reliability.

*Proof.* We start by decomposing σw2* into $\sigma_{w}^{2}$ and $\sigma_{\varepsilon }^{2}$ (see Eq. 12), and derive:

(16) α⁢σw2*=σw2σw2+σε2⁢(σw2+σε2)

which can be rewritten as:

(17) α⁢σw2*=(σw4+σε2⁢σw2)σw2+σε2

and yields:

(18) α⁢σw2*=σw2⁢σw2+σε2σw2+σε2=σw2

This completes the proof.

Theorem 2 (using the sample analogs) can be used to recover an ICC(1) estimation, robust to measurement error. Since we correct the within-variance by reliability, we call the new estimator reliability-adjust ICC(1) estimator, and it is defined as:

(19) ρ^r⁢(y*)=σ^b2σ^b2+α^⁢σ^w2*

where ${\hat{\sigma }}_{b}^{2}$ refers to the estimated between-variance, σ^w2* denotes the estimated biased within-variance (see Eq. 8), $\hat{\alpha }$ denotes the estimated within-reliability of the variable of interest. $\hat{\alpha }$ can be estimated by a *three-level model* (e.g., items nested in events nested in individuals or items nested in individuals nested in groups; see Eq. 21), and is defined as:

(20) $$\hat{\alpha }=\frac{{\hat{\sigma }}_{w}^{2}}{{\hat{\sigma }}_{w}^{2}+\frac{{\hat{\sigma }}_{i}^{2}}{p}}$$

where ${\hat{\sigma }}_{w}^{2}$ represents the within-variance (i.e., level 2 variance; for a definition, see Eq. 24 later in the article), ${\hat{\sigma }}_{i}^{2}$ represents the variance of the items (i.e., level 1 variance; for a definition, see Eq. 22 later in the article), and *p* represents the number of the items used to measure the constructs (e.g., Raudenbush et al., 1991; Nezlek, 2017). We want to emphasize that the formula for the within-variance for the two-level model and for the three-level model are *not* the same (compare Eq. 3 and Eq. 24). Now, all elements of the reliability-adjusted ICC(1) are defined, and it can be estimated from a sample.

The reliability-adjusted ICC(1) estimator corrects the within-variance, by applying Theorem 2, and is therefore robust to measurement error. We can derive Corollary 2.

**Corollary 2** Let *y*^∗^ be a random variable, consisting of a random variable *y* with two levels (e.g., Eq. 1) and some measurement error, ε, then the reliability-adjusted ICC(1) estimator of variable *y*^∗^, ${\hat{\rho }}_{r}\,\left(y^{{}^{*}}\right)$, equals the ICC(1) estimator of variable *y*, $\hat{\rho }\,\left(y\right)$.

ρ^r⁢(y*)=σ^b2σ^b2+α^⁢σ^w2*=ρ^⁢(y)=σ^b2σ^b2+σ^w2

Corollary 2 highlights that the reliability-adjusted ICC(1) estimator applied to variable *y*^∗^ with measurement error is equal to the ICC(1) estimator applied to variable *y* (without measurement error). Accordingly, the reliability-adjusted ICC(1) estimator can be applied to variable with measurement error, and still yields correct estimates for the ICC(1).

## The Measurement Model-Based ICC(1)

As an alternative, we show that the ICC(1) can also be correctly estimated based on a measurement model, which is usually more straightforward for researchers. The measurement model adds the item-level to the original model defined in Eq. 1; the construct’s items are nested in occasions, which in turn, are nested in individuals (i.e., a three-level model; see Eq. 21).

### A Three-Level Measurement Model

Let *y*_*ijk*_ be a random variable with three-levels:

$$y_{i\,j\,k}=\gamma_{00}+U_{j}+u_{i\,j}+\varepsilon_{i\,j\,k}\;i=1,\ldots ,m,j=1,\ldots ,n,$$

(21) k=1,…,l

In this model, **γ**_*00*_, *U*_*j*_, and *u*_*ij*_ were denoted above at Eq. 1, and **ε**_*ijk*_ represents the item specific deviation from *u*_*ij*_ of the item *k* in event *i* in person *j* (i.e., the measurement error). According to the classical measurement error assumptions, the variable **ε**_*ijk*_ is independently normally distributed with a mean of zero and a variance of $\sigma_{\varepsilon }^{2}$ (Lord and Novick, 2008).

We can now define the item-level variance, the within-variance and the between-variance. The estimated variance of the item-level, ${\hat{\sigma }}_{i\,\left(t\,e\,m\right)}^{2}$, is defined as:

(22) $${\hat{\sigma }}_{i}^{2}=\frac{1}{n\,m\,l-n\,m}\,\sum_{j=1}^{n}\sum_{i=1}^{m}\sum_{k=1}^{l}{\left(y_{i\,j\,k}-{\bar{y}}_{j\,i}\right)}^{2}$$

where *n* refers to the number of individuals, *m* refers to the number of occasions per individual, *l* refers to the number of items, and $\bar{y}$_*ij*_ denotes the occasion mean.

$S_{w}^{2}$ is the estimated within-variance (i.e., second level), and defined as:

(23) $$S_{w}^{2}=\frac{1}{n\,m-n}\,\sum_{j=1}^{n}\sum_{i=1}^{m}{\left({\bar{y}}_{i\,j}-{\overset{=}{y}}_{j}\right)}^{2}$$

where $\overset{=}{y}j$ denotes the person mean^6^. $S_{w}^{2}$ contains sampling error (according to the law of summing normally distributed variances; Searle, 1995). A correct estimate of the within-variance is:

(24) $${\hat{\sigma }}_{w}^{2}=S_{w}^{2}-\frac{{\hat{\sigma }}_{i}^{2}}{l}$$

The estimated between-variance is defined as:

(25) $$S_{b}^{2}=\frac{1}{n-1}\,\sum_{j=1}^{n}{\left({\overset{=}{y}}_{j}-\overset{\equiv }{y}\right)}^{2}$$

where $\overset{\equiv }{y}$ denotes the grand mean. $S_{b}^{2}$ contains sampling error (according to the law of summing normally distributed variances; Searle, 1995). To obtain a correct estimate for $\sigma_{b}^{2}$, we must correct $S_{b}^{2}$ for it:

(26) $${\hat{\sigma }}_{b}^{2}=S_{b}^{2}-\frac{S_{w}^{2}}{m}$$

As outlined above, the estimated between-variance and the estimated within-variance of the three-level model can be smaller than zero, potentially. We have now completely defined the variance components of the measurement model.

### The Effect of Measurement Error on Measurement Model-Based ICC(1) Estimator

Since we defined all variance components, we can now prove that measurement error does not affect the estimated within-variance and the estimated between-variance for the three-level model.

We start by showing that measurement error does not induce bias in the within-variance. Creating the occasion mean induces sampling error in the within-variance, as the items are averaged. The variance of ${\bar{y}}_{ij}$ consists of four parts: the constant **γ**_*00*_, the variable *U*_*j*_, the average of the variable *u*_*ij*_ and the average of the variable **ε**_*ijk*_.

(27) $${\bar{y}}_{i\,j}=\frac{1}{l}\,\sum_{k=1}^{l}\left(\gamma_{00}+U_{j}+u_{i\,j}+\varepsilon_{i\,j\,k}\right)=\gamma_{00}+U_{j}+u_{i\,j}+\frac{1}{l}\,\sum_{k=1}^{l}\varepsilon_{i\,j\,k}$$

As the variables **γ**_*00*_, *U*_*j*_, *u*_*ij*_, and **ε**_*ijk*_ are independent, we can write the variance of ${\bar{y}}_{ij}$ as the sum of their variances.

$$V\,a\,r\,\left({\bar{y}}_{i\,j}\right)=V\,a\,r\,\left(\gamma_{00}\right)+V\,a\,r\,\left(U_{j}\right)+V\,a\,r\,\left(u_{i\,j}\right)+V\,a\,r\,\left(\frac{1}{l}\,\sum_{k=1}^{l}\varepsilon_{i\,j\,k}\right)$$

(28) $$={\hat{\sigma }}_{w}^{2}+\frac{{\hat{\sigma }}_{i}^{2}}{l}$$

*Var*(*γ*_00_) and *Var*(*U*_*j*_) are constants for a chosen *j*, therefore their variance is zero. Eq. 28 shows that the variance of ${\bar{y}}_{ij}$ equals the within-variance, ${\hat{\sigma }}_{w}^{2}$, plus the sampling error $\frac{{\hat{\sigma }}_{i}^{2}}{l}$. As the estimated within-variance (see Eq. 24) is corrected for sampling error, it is unaffected by measurement error.

Further, creating the person mean induces sampling error in the between-variance, as the occasions and items are averaged. The variance of ${\overset{=}{y}}_{j}$ consists of four parts: the constant **γ**_*00*_, the variable *U*_*j*_, the average of the variable *u*_*ij*_ and the average of the variable **ε**_*ijk*_.

$${\overset{=}{y}}_{j}=\frac{1}{m}\,\sum_{i=1}^{m}\left(\gamma_{00}+U_{j}+u_{i\,j}+\frac{1}{l}\,\sum_{k=1}^{l}\varepsilon_{i\,j\,k}\right)$$

(29) $$=\gamma_{00}+U_{j}+\frac{1}{m}\,\sum_{i=1}^{m}u_{i\,j}+\frac{1}{m}\,\sum_{i=1}^{m}\frac{1}{l}\,\sum_{k=1}^{l}\varepsilon_{i\,j\,k}$$

As the variables **γ**_*00*_, *U*_*j*_, *u*_*ij*_, and **ε**_*ijk*_ are independent, we can write the variance of ${\overset{=}{y}}_{j}$ as the sum of their variances.

$$V\,a\,r\,\left({\overset{=}{y}}_{j}\right)=V\,a\,r\,\left(\gamma_{00}\right)+V\,a\,r\,\left(U_{j}\right)+V\,a\,r\,\left(\frac{1}{m}\,\sum_{i=1}^{m}u_{i\,j}\right)+$$

(30) $$V\,a\,r\,\left(\frac{1}{m}\,\sum_{i=1}^{m}\frac{1}{l}\,\sum_{k=1}^{l}\varepsilon_{i\,j\,k}\right)={\hat{\sigma }}_{b}^{2}+\frac{{\hat{\sigma }}_{w}^{2}+\frac{{\hat{\sigma }}_{i}^{2}}{l}}{m}$$

*Var*(*γ*_00_) is zero. Eq. 30 shows that variance of ${\bar{y}}_{ij}$ equals the between-variance, ${\hat{\sigma }}_{b}^{2}$ plus sampling error of level 1 and level 2, $\frac{{\hat{\sigma }}_{w}^{2}+\frac{{\hat{\sigma }}_{i}^{2}}{l}}{m}$. The sampling error equals $\frac{S_{w}^{2}}{m}$. As the estimated between-variance (see Eq. 26) is corrected for sampling error, it is unaffected by measurement error. As the within-variance (see Eq. 24) and between-variance (see Eq. 26) are corrected for sampling error, we conclude that the within-variance and the between-variance of a measurement model are not affected by measurement error. This completes the proof. We conclude that the measurement model-based ICC(1) estimator yields estimates, unaffected by measurement error, and can be estimated by Eq. 31.

### The Estimation of the Measurement Model-Based ICC(1)

The measurement model-based ICC(1) estimator is based on the measurement model, and defined as:

(31) $${\hat{\rho }}_{m}\,\left(y\right)=\frac{{\hat{\sigma }}_{b}^{2}}{{\hat{\sigma }}_{b}^{2}+{\hat{\sigma }}_{w}^{2}}$$

where ${\hat{\sigma }}_{b}^{2}$ denotes the estimated between-variance and ${\hat{\sigma }}_{w}^{2}$ denotes the estimated within-variance of a three-level model. It is important to note that the definition of the measurement model-based ICC(1) is different to the normal definition of the ICC(1) for a three-level model, as the measurement model-based ICC(1) does not divide by the total-variance, but just by the sum of the between-variance and within-variance.

## Discussion

The present article examined the effect of measurement error on ICC(1) estimation, and presented two estimators – the reliability-adjusted ICC(1) and the measurement model-based ICC(1) – that yield ICC(1) estimates, corrected for measurement error.

### The Effect of Measurement Error on ICC(1) Estimation

The first part of this study examined the effect of measurement error on ICC(1) estimation. The presence of non-negligible measurement error variance induces positive bias to the within-variance estimation (see Theorem 1), when built with the standard formulas (see Eq. 3). It is clear from Theorem 1 that the bias in the ICC(1) is driven by the variance of the measurement error, which increases the within-variance expression, resulting in overestimation of the correct within-variance value. Consequently, the positive bias in the within-variance estimate increases the denominator of the ICC(1) expression, which in turn leads to an underestimation of the ICC(1) expression (see Corollary 1). In other words, the less negligible the variance of the measurement error, the higher the estimated within-variance, and the lower the estimated ICC(1).

In order to illustrate the effect of measurement error on ICC(1) estimation, we created a numerical example, which compares the RMSE, the alpha error rate and the inclusion of zero in the confidence interval for ICC(1) estimation with and without measurement error. The numerical example must be interpreted in light of acceptable values of reliability in the field of psychology. Shrout (1998, p. 308) provided guidance for this: “0.00–0.10, virtually no reliability; 0.11–0.40, slight; 0.41–0.60, fair; 0.61–0.80, moderate; 0.81–1.0, substantial.” For intraindividual studies, Nezlek (2017) even suggests somewhat more relaxed standards. We assume that researchers, and reviewers alike, probably accept reliabilities of 0.9 and 0.7, as they are considered moderate with the recommended rule of thumbs (Shrout, 1998; Nezlek, 2017). A reliability of 0.5 also appears to be likely to be accepted in intraindividual studies, as many constructs are measured only with very few or even a single item to reduce the burden of the participants (e.g., Bolger et al., 2003). Based on Corollary 1 and our numerical example, we conclude that measurement error, firstly, decreases – slightly to severely – many of the reported ICC(1) estimates, secondly, misleads inference based on the ICC(1) estimator, and thirdly, may falsely yield ICC(1) estimates, indicating independent data in the literature of psychology.

### The Reliability-Adjusted ICC(1) and the Measurement Model-Based ICC(1)

In the second part of the present article, we proposed two corrections for ICC(1) estimation, robust to measurement error.

We firstly derived the reliability-adjusted ICC(1). According to Theorem 2, the product of the estimated reliability Eq. 20 and the measurement error-affected within-variance (see Eq. 8) of the same construct equals the actual within-variance of the construct (see Eq. 3). In other words, if we constrain the measurement error-affected within-variance to the fraction of error free variance, we obtain the within-variance, unaffected by measurement error. Making use of Theorem 2, we derived the formula for ICC(1) estimation, unaffected by measurement error (see Eq. 19). Measurement error does not induce bias in the reliability-adjusted ICC(1) estimator.

Secondly, we derived the measurement model-based ICC(1). We have proven that measurement error does not affect the within-variance, ${\hat{\sigma }}_{w}^{2}$, of a measurement model, when estimated with the standard formula (see Eq. 24). But, the items contain measurement error, and when they are aggregated to reflect the mean of the items, their aggregation induces sampling error (see Eq. 28). As the standard formula for the within-variance, ${\hat{\sigma }}_{w}^{2}$, corrects for sampling error, the within-variance of the measurement model remains unaffected by measurement error (see Eq. 24). The same holds true for the between-variance (see Eq. 26 and Eq. 30). Accordingly, the measurement model-based ICC(1) is robust to measurement error. It is important to note that the measurement model-based ICC(1) divides the between-variance by the sum of the between-variance and the within-variance, but not by the total-variance. The variance of the item-level must not be included in the total variance, as it represents variance due to measurement.

The reliability-adjusted ICC(1) and the measurement model-based ICC(1) yield essentially the same result. However, the measurement model-based ICC(1) estimator requires to have access to the raw data in order model the different levels. By contrast, the reliability-adjusted ICC(1) estimator only needs the ICC(1) estimates and the estimate of the reliability of the construct of interest, and it can be calculated. The ICC(1) estimate reflects a proportion that remains constant, when each term is scaled by constant (i.e., $\frac{{\hat{\sigma }}_{b}^{2}}{{\hat{\sigma }}_{b}^{2}+{\hat{\sigma }}_{w}^{2}}$ = $\frac{c\,{\hat{\sigma }}_{b}^{2}}{c\,{\hat{\sigma }}_{b}^{2}+c\,{\hat{\sigma }}_{w}^{2}}$). Accordingly, we can apply the reliability-adjusted ICC(1) estimator to any ICC(1) estimate found in the literature without knowing the exact within-variance or between-variance of the construct of interest. It is just necessary to find values that are proportional to the between-variance and within-variance, and in combination equal the found ICC(1) estimate. The reliability estimate can then be used to correct the proportional within-variance, which in turn can be used to yield the reliability-adjusted ICC(1). Therefore, the reliability-adjusted ICC(1) may be particularly useful for meta-analyses, as the raw data is only seldomly used (or available), but the estimated reliabilities may be available instead. In those cases, the reliability-adjusted ICC(1) is more useful than the measurement model-based ICC(1) estimator.

To give examples, why it is important to correct for measurement error, and thus use the proposed estimators, we refer back to our examples from the introduction. Podsakoff et al. (2019) examined ICC(1) for 23 different constructs (e.g., personality, coping, and job characteristics) from 222 intraindividual studies, and concluded that many psychological constructs have at least about 40% and the majority of constructs more than 50% of within-variance. Based on Theorem 1, we must assume that psychological constructs probably vary less within individuals than their meta-analysis suggests, as it is not common practice to correct the within-variance of the ICC(1). Further, if we are willing to assume a reliability, we can also estimate a corrected within-variance with Theorem 2. In case of a reliability of 0.5 (0.7), many psychological constructs have at least about 25% (32%) and the majority of constructs more than 33% (41%) of within-variance. Accordingly, if we correct for measurement error, we conclude differently about the within-variability of psychological constructs.

Likewise, Kivlighan et al. (2019) examined 169 effect sizes from 37 group treatment studies, stressed group therapy members *mutually influence* their group posttreatment outcomes (e.g., depression, etc.), and found that group membership explains about 6% of variance [ICC(1) = 0.06; Cohen’s *d* of about 0.47]. Based on Corollary 1, we must assume that they underestimated the effect size of mutual influence. If we are willing to assume a reliability, we can also estimate a corrected ICC(1) based on Corollary 2. In case of a reliability of 0.9 (0.7), reliability-adjusted ICC(1) is 0.07 (0.08). If we now also assume that the standard error does not change after the correction, we can also correct their effect size to a Cohen’s *d* of 0.52 (0.65). Accordingly, if we correct for measurement error, we conclude that mutual influence is more important than originally claimed by Kivlighan et al. (2019).

Further, we want to examine the inclusion of zeros in the confidence interval. The ICC(1) is often used to determine non-dependence or dependence of hierarchical data (Bliese, 2000). The data is non-dependent, if the ICC(1) is essentially zero. If the confidence interval includes zero, the ICC(1) is not essentially different from zero. Our numerical example revealed that the combination of large measurement error (i.e., reliability of 0.7 or 0.5), a population ICC (1) equal to 0.1, and small sample sizes on level 1 (i.e., *m* = 4) may pose a severe threat to the understanding whether data is dependent or independent. In those cases, the ICC(1) estimate, affected by measurement error is not essentially different from zero about 24% (reliability of 0.7) or 50% (reliability of 0.5) of the times, but the ICC(1) estimate, unaffected by measurement error only about 5 or 6% of the times. If the ordinary least squares estimator is applied to dependent data, resulting standard errors may be extremely biased and inference may be flawed (e.g., Wampold and Serlin, 2000; Musca et al., 2011). Accordingly, if the ICC(1) is used to determine dependency in the data, the adjusted estimators should be used to avoid the risk of biased standard errors in dependent data.

Finally, our work can be applied to other ICCs. Generally, different ICCs can be defined as the ratio of the variance of interest and the total variance (Liljequist et al., 2019). Theorem 1 shows that the within-variance has a positive bias under condition of measurement error, which may be important to other ICCs that use the within-variance (e.g., as part of the total variance). Further, if they want to cancel the measurement error-variance, they can apply Theorem 2 to derive an estimator for ICC of interest, unbiased by measurement error.

### Limitations and Future Research

Some limitations and directions for future research should be noted here.

Firstly, the current study did not derive the formula for the confidence intervals for the reliability-adjusted ICC(1) estimator and the measurement model-based ICC(1) estimator. It will be important to derive them in future research, as confidence intervals are required to make inference with the estimators of ICC(1), robust to measurement error, such as hypothesis testing (e.g., Kivlighan et al., 2019) or determining dependence in data (e.g., Bliese, 2000).

Secondly, the current study solely relied on normally distributed between-effects and within-effects, and did not examine other distributions. For example, a typical reliability measure, the Cronbach’s alpha (Sheng and Sheng, 2012), is biased, given non-normal data. Therefore, the ICC(1), robust to measurement error may also be affected by non-normal data. Additionally, the behavior of the proposed estimators may change under condition of different missing data patterns (e.g., Shieh, 2012). We hope that future research addresses these limitations of the present study.

Finally, the Bayesian approaches to ICC(1) estimation (e.g., Zhang and Wang, 2018; Mulder and Fox, 2019) may suffer from the same problem of measurement error as the frequentist approach, as Theorem 1 should hold, independent of the estimation approach. Therefore, future research could develop Bayesian equivalents to the reliability-adjusted ICC(1) estimator and the measurement model-based ICC(1) estimator.

## Conclusion

All in all, we conclude that measurement error induces a non-negligible downward bias in ICC(1) estimation, as shown in the example of Kivlighan et al. (2019) and Podsakoff et al. (2019). Our proposed estimators – the reliability-adjusted ICC(1) estimator and measurement model-based ICC(1) estimator – yield estimates, corrected for measurement error. We hope that our proposed estimators will help researchers to obtain a more accurate ratio of between-variance to total-variance in the future under the condition of measurement error.

## Data Availability Statement

All datasets generated for this study are included in the article/Supplementary Material.

## Author Contributions

RW designed the concept of manuscript, wrote the technical derivations, created the simulations, and drafted the manuscript. RL and AK contributed substantially in the process by providing feedback at all stages of the manuscript. All authors reviewed and commented on drafts of the manuscript and read and approved the final manuscript.

## Conflict of Interest

The authors declare that the research was conducted in the absence of any commercial or financial relationships that could be construed as a potential conflict of interest.

## Footnotes

1 The ICC shows up in two distinct areas in statistics: reliability and clustered data. We refer to the ICC in the area of clustered data (e.g., Raudenbush et al., 1991; Snijders and Bosker, 2012).

2 The lower the variance of the measurement error, the higher the reliability, and vice versa (e.g., Lord and Novick, 2008).

3 ICCs can be defined in various ways, given there are more than two-levels (e.g., McGraw and Wong, 1996; Baguley, 2012).

4 Shieh (2012) notes that truncating the between-variance induces positive bias, in particular when the population ICC(1) small.

5 In the Supplementary Appendix 3, we provide a practical guide on how to adjust the ICC for measurement error in *R Cran* (R Core Team, 2015) with the package *lmer* (Bates et al., 2015).

6 The estimators of the grand mean and the person mean are defined in Supplementary Appendix 2.
