# Supplementary material for: Do We Overestimate the Within-Variability? The Impact of Measurement Error on Intraclass Coefficient Estimation
Source: Front Psychol. 2020 May 19;11:825. doi: 10.3389/fpsyg.2020.00825 (PMC7248308; doi:10.3389/fpsyg.2020.00825)
Supplement: Supplementary file 1 [file Presentation_1.pdf]

Appendix

Appendix 1: Definitions of the means of a two-level model.

The grand mean (e.g., Snijders and Bosker, 2012) represents the mean among all observations, and is defined as:

$$\bar{\bar{y}} = \frac{1}{N}\sum_{i=1}^{n}\sum_{j=1}^{m} y_{ij},$$

where *N* refers to the total number of observations in the data set.

The person mean represents the mean of person j, and is defined as:

$$\bar{y}_j = \frac{1}{m}\sum_{i=1}^{m} y_{ij},$$

where *m* refers to the number of observations for person *j*. $\bar{y}_j$ represents all the person means from individual 1 to individual *j* (e.g., Snijders and Bosker, 2012).

Appendix 2: Definitions of the means of a three-level model

The grand mean (e.g., Snijders and Bosker, 2012) is defined as:

$$\bar{\bar{\bar{y}}} = \frac{1}{nml}\sum_{i=1}^{n}\sum_{j=1}^{m}\sum_{k=1}^{l} y_{ijk},$$

where *n* represents the number of individuals, *m* represents the number of occasions, and *l* represents the number of items.

The person mean (e.g., Snijders and Bosker, 2012) is defined as:

$$\bar{\bar{y}}_j = \frac{1}{ml}\sum_{i=1}^{m}\sum_{k=1}^{l} y_{ijk}.$$

The occasion mean (e.g., Snijders and Bosker, 2012) for person *j* in occasion *i* is defined as:

$$\bar{y}_{ji} = \frac{1}{l}\sum_{k=1}^{l} y_{ijk}$$

Appendix 3: A practical guide to estimate the ICC correctly

In this section, we present how to estimate, firstly, the reliability-adjusted ICC(1) and, secondly, the measurement model-based ICC(1) with the package lmer (Bates et al., 2015) in R Cran (R Core Team, 2015) conveniently. Since their calculation partially overlaps, we first explain the data structure, the syntax, and the output of a two-level model and a three-level model in general, and then use their outputs to calculate the respective ICC(1).

Data structures

The two-level model data structure

The two-level model data structure (See Table 1) contains a column indicating the individuals ID, a column indicating the occasion, and a column indicating the averaged value of the items of the focal construct. The first row indicates that the person with ID 1 has an average y value of 1.5 on occasion 1. Note that each individual must have a unique ID.

Table 1: Exemplary data structure for a two-level model

Row number	ID	Occasion	$\bar{y}$
1	1	1	1.5
2	1	2	1.5
3	2	1	4.5
4	2	2	3.5

The three-level model data structure

The three-level model data structure (See Table 2) contains a column indicating the individuals ID, a column indicating the occasion, a column indicating the item's ID, and a column indicating the value of person *j* in occasion *i* in item k. The first row indicates that the person with ID 1 has a y value of 1 in occasion 1 on item 1. Note that the individual's IDs and the occasion's ID must be unique.

Table 2: Exemplary data structure for a three-level measurement model

Index	ID	Occasion	Item	y
1	1	1	1	1
2	1	1	2	2
3	1	2	1	2
4	1	2	2	1
5	2	3	1	4
6	2	3	2	5
7	2	4	1	6
8	2	4	2	1

Syntax

Syntax for a two-level unconditional model

For the two-level data structure, we run a two-level unconditional model. An unconditional model only contains the means for the different levels. For a two-level model, it only contains the means for each individual observed. The syntax is:

```
> library(“lmer”)

> Model1 <- lmer(y ~ (1|ID), data.file)

> summary(Model1)
```

The first row of the statement library(“lmer”) loads the package lmer (Bates et al., 2015). The second row begins with an arbitrary model name. Here we chose Model1, but any useful model name works. To the right of the model name, “<-“ represents the assignment operator, which assigns the output of a function to an object. The model name refers to this object, which is stored in memory and can be called later by its name. The function lmer is R’s multilevel implementation (Bates et al., 2015). It begins with an opening parenthesis, which is followed by the dependent variable of the model, y. Next, the tilde (~) denotes regress on. A 1 after a tilde includes an intercept in the model. After the

second opening parentheses follows the random effect on the individual level, ID. The random effect has the form (1|ID) and indicates that the intercepts vary across IDs. In other words, each individual obtains an intercept. The variance of these intercepts equals the between-variance. The closing parenthesis closes the statement for the random effect on the individual level. The last part of the statement indicates the used data-file, which is separated by a comma. Finally, the last parenthesis closes the statement.

In the third row, the summary command prints the results of the two-level.

Syntax for a three-level unconditional model

For the three-level data structure, we run a three-level unconditional model with the syntax:

```
> library("lmer")

> Model2 <- lmer(y ~ (1|Occasion) + (1|ID), data.file)

> summary(Model2)
```

Since the syntax for the three-level model is similar to the two-level model, we only explain the differences between them. The second row contains two random intercepts, (1|Occasion) and (1|ID). In other words, not only each individual obtains an intercept, but also each occasion. The variance of the individual's intercepts equals the between-variance. The variance of the occasion's intercepts equals the within-variance.

Output

Output for a two-level unconditional model

Table 3 shows the output of Model1 (i.e., the two-level unconditional model).

Table 3: Exemplary summary of the two-level unconditional model

Random effects			
Groups	Name	Variance	Std.Dev.
ID	(Intercept)	0.979	0.989
Residual		1.472	1.213

It contains information about the groups, random intercepts, variance and standard deviation. The column Groups indicates that the summary of the model contains information about the predictor ID and the residuals. The column Name indicates here which of these rows refers to a random intercept. The rows Variance and Std.Dev. contain information about the variance and standard deviation,

respectively. The variance of ID, the between-variance, equals 1.016, whereas variance of Residual, the within-variance, equals 1.324.

Output for a three-level unconditional model

Table 4 shows the output of Model2 (i.e., the three-level unconditional model).

Table 4: Exemplary summary of the three-level unconditional model

Random effects			
Groups	Name	Variance	Std.Dev.
Occasion	(Intercept)	0.975	0.988
ID	(Intercept)	0.979	0.989
Residual		0.993	0.997

The output of the three-level unconditional model is very similar to Table 3, except that it contains two random intercepts (i.e., ID and Occasion). The variance of ID, the between-variance, equals 1.016, variance of Occasion, the within-variance, equals 0.998, and the variance of Residual, the measurement error's variance, equals 0.978.

The proposed estimators of the ICC(1), robust to measurement error

The reliability-adjusted ICC(1)

The calculation of the reliability-adjusted ICC(1) requires to estimate, firstly, the uncorrected ICC(1) and, secondly, the reliability on the within-level (e.g., Raudenbush et al., 1991; Lord and Novick, 2008; Bonito et al., 2012). The estimated ICC(1) is based on the two-level model, and can be calculated by **Error! Reference source not found.**, where the between-variance (i.e., the row ID) and within-variance (i.e., the row Occasion) are displayed in Table 3. In the current example, the unadjusted ICC equals 0.399, whereas the reliability-adjusted ICC equals 0.501[1].

The measurement model-based ICC(1)

The measurement model-based ICC(1) is based on the three-level model, and can be calculated by **Error! Reference source not found.**, where the between-variance (i.e., the row ID) and within-variance (i.e., the row Occasion) are displayed in Table 4. In the current example, the measurement model-based ICC equals 0.501. The reliability-adjusted ICC and the measurement model-based ICC are equivalent.

[1] The reliability equals 0.663
